# Using natural language processing in facilitating pre-hospital telephone triage of emergency calls

**DOI:** 10.29045/14784726.2022.09.7.2.31

**Published:** 2022-09-01

**Authors:** Kevin Gormley, Katy Lockhart, Jolly Isaac

**Affiliations:** Mohammed Bin Rashid University of Medicine and Health Sciences; NHS Digital; Mohammed Bin Rashid University of Medicine and Health Sciences

**Keywords:** cardiac arrest, emergency calls, natural language processing, triage

## Abstract

**Introduction:**

Natural language processing (NLP) is an area of computer science that involves the use of computers to understand human language and semantics (meaning) and to offer consistent and reliable responses. There is good evidence of significant advancement in the use of NLP technology in dealing with acutely ill patients in hospital (such as differential diagnosis assistance, clinical decision-making and treatment options). Further technical development and research into the use of NLP could enable further improvements in the quality of pre-hospital emergency care. The aim of this literature review was to explore the opportunities and potential obstacles in implementing NLP during this phase of emergency care and to question if NLP could contribute towards improving the process of nature of call screening (NoCS) to enable earlier recognition of life-threatening situations during telephone triage of emergency calls.

**Methods:**

A systematic search strategy using two electronic databases (CINAHL and MEDLINE) was conducted in December 2021. The PRISMA systematic approach was used to conduct a review of the literature, and selected studies were identified and used to support a critical review of the actual and potential use of NLP for the call-taking phase of emergency care.

**Results:**

An initial search offered 204 records: 23 remained after eliminating duplicates and a consideration of title and abstracts. A further 16 full-text articles were deemed ineligible (not related to the subject under investigation), leaving seven included studies. Following a thematic review of these studies two themes emerged, that are considered individually and together: (i) use of NLP for dealing with out-of-hospital cardiac arrest and (ii) responding to increased accuracy of NLP.

**Conclusions:**

NLP has the potential to reduce or eliminate human bias during the emergency triage assessment process and contribute towards improving triage accuracy in pre-hospital decision-making and an early identification and categorisation of life-threatening conditions. Evidence to date is mostly linked to cardiac arrest identification; this review proposes that during the call-taking phase NLP should be extended to include further medical emergencies (including fracture/trauma, stroke and ketoacidosis). Further research is indicated to test the reliability of these findings and a proportionate introduction of NLP simultaneous with increased quality and reliability.

## Introduction

Emergency calls to ambulance services nationally and internationally are generally triaged using clinical decision support systems, and calls are prioritised by trained individuals (call handlers) to determine appropriate and proportionate responses (Blomberg et al., 2019; Bresnick, 2018). At the beginning of a call, call handlers determine and insert a tag for the emergency into the computer-aided dispatch system which forms the beginning phase of prioritising and allocating resources to the dispatch team. The next stage involves a pre-triage sieve of nature of call screening (NoCS) when the caller is invited to provide a summary of the emergency and answer agreed questions. This typically involves an open narrative including a description of the personal and clinical circumstances from the caller; the call handler, on the basis of responses, chooses from a pre-set selection of actions (Bresnick, 2018). Green et al. (2019) described how call handlers are able to recognise and identify immediate threats to life and request the chief option (CO) response: a call-screening tool that establishes a need for an immediate dispatch of ambulance to an out-of-hospital cardiac arrest (OHCA).

Natural language processing (NLP) is an area of computer science involving the use of computers to understand human language and its semantics (meaning) and provide consistent and reliable responses (LeCun et al., 2015; Muller & Brejnebøl, 2019; Oubenali et al., 2022). NLP is a part of daily life, for example commercial products such as Amazon’s Alexa and Apple’s Siri provide useful daily living examples of the use of this form of technology (Deakin et al., 2017; NHS England, 2018).The expectation is that NLP should enable computers to process and understand human language through reading, translating and clarifying interpretation, and through this process make logical and constructive sense of the information provided and consistently and reliably respond (Garbade, 2018).

If this can be achieved, it is conceivable and appropriate that health-caring services should make use of NLP to engage in patient assessment and triage decisions, particularly during pre-hospital emergency situations. It is understandably difficult for some to appreciate the concept of discussing emergency situations and care, including presenting signs and symptoms, through NLP but in practice it has already generated a constructive and reasonably balanced discussion. The idea that screening and option decision-making could be undertaken, at least in part, through NLP forms the purpose and rationale for this review. Irojou and Olaleke (2015) in a systematic review of NLP and emergency healthcare identified early terminology issues, spelling and pronunciation difficulties. Although important, the authors also pointed out that these issues already exist among call handlers through different local accents and use of language.

Wang et al. (2020) concurred and contended that as the technology continues to develop with increased consistency the weighting of support for NLP during this phase of care will grow. Wang was not proposing that human call handlers should not be used at all but argued for a greater use of NLP, wherever it is evident that NLP can positively enhance and improve clinical decision-making in the emergency care setting. The aim of this literature review was to explore the opportunities and potential obstacles in implementing NLP during this phase of emergency care and it questioned if NLP could contribute towards improving the process of NoCS to enable earlier recognition of life-threatening situations during telephone triage of emergency calls.

## Study design

The purpose of a literature review is to examine the totality of selected and focused published arguments. For this review, a narrative synthesis design was adopted, offering a robust mechanism where findings can be uniquely viewed interpreted and constructively brought together as key themes of academic interest (Popay et al., 2006; Saltikov, 2012).

## Search strategy

**Figure 1:**
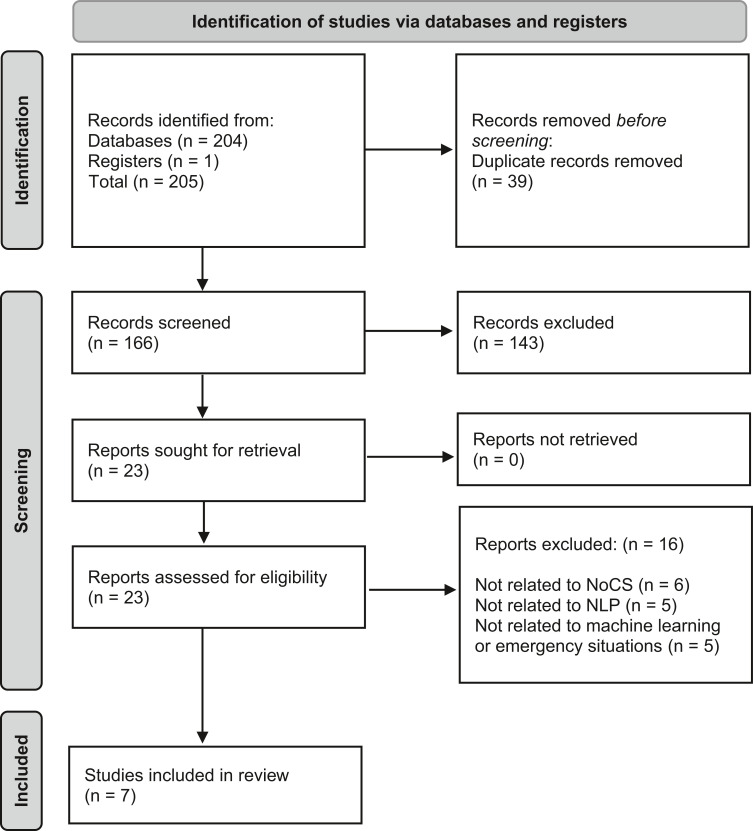
Figure 1. PRISMA flow diagram.

NoCS: nature of call screening; NLP: natural language processing.

Research articles were selected using the Preferred Reporting Items for Systematic reviews and Meta-Analyses (PRISMA) guidelines, which assisted in refining the search and ensured alignment of articles with the review’s focus and purpose (Figure 1). PRISMA is designed to help systematic reviewers report on the reasons for conducting the review and present what was found using appraising and synthesis skills (Page et al., 2021). The search was conducted in December 2021, using CINAHL and MEDLINE electronic databases to identify relevant studies published in English between the years 2012 and 2021. A selection of keywords and mesh terms were used interchangeably to maximise the search return. These included: neural networks, machine learning, deep learning, natural language processing, emergency calls, semantics, nature of call and emergency care. These words and terms were combined using AND and OR conjunctions.

## Study selection

Flexibility was also required by the authors, one of whom is an expert in the field, in the use of terms to ensure appropriate use of synonyms and acronyms (Figure 1). Included studies were required to be published in English and in peer-reviewed journals. Case studies and literature reviews were excluded. Reference lists were also considered for possible studies. Several papers were excluded because they were deemed ineligible (they did not meet the inclusion criteria or examine specifically a feature of NLP and emergency care). 204 records were identified through database searching. After eliminating duplicates, the abstracts and titles of the remaining 166 studies were screened, from which 23 studies were shortlisted. A full-text eligibility analysis was then conducted, from which 16 were eliminated, leaving seven included studies (Figure 1).

## Quality appraisal

Two authors independently assessed and rated each of the seven selected studies using an amended checklist based on Hawker et al.’s (2002) critical appraisal tool. This tool was selected because of its transferable use across different research methodologies (Hawker et al., 2002). The checklist enabled a structured appraisal of the seven studies using nine separate sections (including methods, data, sampling, implications) that were rated on a scale of 0 (poor) to 3 (very good), meaning a maximum possible score of 27 (Table 1).

**Table 1.** Included articles and quality checklist from Hawker et al. (2002).

Author, country and study design	Aims	Sampling and data collection	Findings	Quality approval total score allocation. Max. score +27 (Hawker et al., 2002)
1. Berdowski, et al. (2009), Netherlands, observational study	Investigated the recognition of a cardiac arrest by dispatchers and its influence on survival rates.	Voice recordings of 14,800 high-priority emergency calls	Spontaneous words that the caller uses to describe the patient may aid in faster and better recognition of a cardiac arrest.	25
2. Blomberg et al. (2019), Denmark, retrospective comparative analysis	Examined whether a machine-learning framework could recognise OHCA from audio files of calls to the emergency medical dispatch centre.	Secondary analysis of 108,607 emergency calls	The machine-learning framework performed better than emergency medical dispatchers for identifying OHCA in emergency phone calls.	24
3. Blomberg et al. (2021), Denmark, RCT	Examined how a machine-learning model trained to identify OHCA and alert dispatchers during emergency calls affected OHCA recognition and response.	Sampling of 169,049 emergency calls	No significant improvement in dispatchers’ ability to recognise cardiac arrest when supported by machine learning.	25
4. Green et al. (2019), England, retrospective observational study	Evaluated the accuracy of NoCS and NHSP in identifying patients with potentially treatable or imminent OHCA.	Secondary analysis of 71,373 emergency telephone records	NoCS and NHSP achieved similar sensitivity for the identification of OHCA but using both methods sequentially improved accuracy.	25
5. Ivanov et al. (2021), United States, retrospective comparative study	Aimed to determine whether historical emergency hospital data could be used with clinical NLP and machine-learning algorithms to produce accurate predictive models.	Secondary analysis of 147,052 encounters from medical records	The triage model was more accurate than the triage nurses in the study sample.	26
6. Joseph et al. (2020), United States, retrospective, cross-sectional study	Examined whether deep-learning approaches could identify critically ill patients only using data immediately available at triage.	Secondary analysis of 445,925 medical records	Deep-learning techniques represent a promising method for augmenting triage, even with limited information.	25
7. Tootooni et al. (2019), United States, retrospective survey analysis	Automatically mapped free-text data into a structured list of chief complaints. Developed an NLP-based algorithm, referred to as chief complaint mapper.	Secondary analysis of 78,000 patient emergency calls	A high level of agreement between two physicians, with less than 2% human error. Proposed it could be adopted by other EDs to support clinical decision-making.	25

ED: emergency department; NHSP: NHS Professionals; NLP: natural language processing; NoCS: nature of call screening; OHCA: out-of-hospital cardiac arrest.

## Data extraction and results

The author’s agreed ratings for each study ranged from good (24) to very good (26) and as a result all seven were deemed appropriate for the literature. The key element of a narrative synthesis is the extraction of meaningful information from each study and integrating this information through a unique configuration and transparent interpretation that is auditable and evidently based upon extracted findings from the studies (Saltikov, 2012).Through an agreed process of reading and re-reading each of the studies and synthesising ideas, the authors determined and agreed the existence of two overarching themes: (i) use of NLP for dealing with OHCA and (ii) responding to increased accuracy of NLP.

## Theme 1: use of natural language processing for dealing with out-of-hospital cardiac arrest

In the United Kingdom, emergency calls are dealt with by non-clinical call handlers, differing from dispatch centre models in Denmark (Blomberg et al., 2019), which are clinically led by nurses and paramedics. It might well be the case that NLP with machine learning is more effective for the UK model due to the lack of clinical expertise or conversely it could be argued that emergency services adopt a US model and increase the clinical experience of call handlers and use of NLP. Ivanov et al. (2021) compared the triage acuity performance of a machine-learning model against emergency department (ED) triage nurses. Data were analysed retrospectively to determine whether they could have more accurately been translated. According to the authors, the NLP model was considered more accurate, even with high-risk patients, including those presenting with chest pain, altered loss of consciousness or suicidal thoughts.

NLP has emerged as an option for use in the pre-hospital care environment specifically to help identify OHCA and to assist in point-of-contact clinical decision-making and responses. Two studies found that the time-to-recognition of cardiac arrest was quicker by an average of 10 seconds for the NLP framework compared to that of dispatchers (Blomberg et al., 2019, 2021). Other studies reviewed the survival rate among cardiac arrest patients identified through ambulance service dispatchers and concurred that the effective NoCS and early and accurate recognition of indicative emergency signs and symptoms correlate with reduced mortality rates (Green et al., 2019; Muller & Brejnebøl, 2019). Joseph et al. (2020) revealed that using NLP can identify critically ill patients using the initial triage to detect the patient’s primary symptoms; the study went on to report much higher accuracy than conventional triage approaches. In contrast, Blomberg et al. (2021) observed NLP in a live emergency call centre and indicated no significant improvement in the recognition of OHCA between dispatcher and machine learning.

Berdowski et al. (2009) considered the use of triggering words used by callers, arguing that certain words occurred more frequently during OHCA calls compared with other life-threatening emergency calls. Green et al. (2019) proposed that the use of trigger terms by callers should also be used to confirm the need for a CO and proposed the use of a NoCS tool to identify OHCA. Tootooni et al. (2019) also called for prompt and reliable use of the CO, contending that informed decision-making should begin at the point of NoCS and as more data are obtained from the caller through initial triage this should be utilised to enable either the activation of the CO or descaling to a more appropriate option level.

According to Green et al. (2019), an NoCS screening tool had similar levels of sensitivity and accuracy for identifying OHCA as pre-existing triage call tools. When both tools were used together, however, a noted and improved accuracy for detecting OHCA with prompt ambulance dispatch was identified; this suggests some benefits for a conjoint approach between NLP and dispatchers. Overall, there was general agreement that ED triage was most accurate when machine learning was utilised (Jiang et al., 2017; Tootooni et al., 2019). It was further concluded (Joseph et al., 2020) that the use of machine learning within ED was also likely to significantly improve the triage process.

## Theme 2: responding to increased accuracy of natural language processing

The value of NLP is the capacity to extract useful information from unstructured narratives that standard machine learning cannot recognise (Irojou & Olaleke, 2015). This happens through the identification of keywords that assist with clinical decision-making. Clearly this is beneficial in improving the quality of services. Nevertheless, there remains the issue of inadequate appreciation of the variation in the use of keywords, which can lead to inaccuracies, and performance issues such as neglecting or ignoring words or just difficulty in interpreting words or terms (Tootooni et al., 2019). Differences were also reported between clinicians in creating set lists, and the fixed structure of these lists was the cause of substantial disparities in human judgement. There was also a need to improve performance refinements (NHS England, 2018; Tootooni et al., 2019).

It was also suggested that to ensure maximum accuracy, variations of keywords should be minimised and captured in the NLP algorithm. In the United Kingdom, a good example of variations in the interpretation of words would be the clinical features of life-threatening shock. In some parts of the country, the term ‘clammy’ is understood to be a cold sweat but in others, hot and sweaty. It will be a challenge in these circumstances for machine learning to interpret these differences and ensure local and national accuracy. Further studies describe how important it is for NLP to be piloted and validated in different locations such as urban and rural along with different patient age and cultural groups (Ivanov et al., 2021; Tootooni et al., 2019). Blomberg et al. (2019, 2021) raised the potential of NLP being able to identify immediate life-threatening situations other than cardiac arrest, such as stroke, acute myocardial infarction or sepsis.

Jiang et al. (2017) pointed out that NLP should have the capacity to recognise principal signs of symptoms that can be present following a stroke in terms of early identification, improved accuracy and greater reliability in measuring and recognising key detecting indicators. Acute stroke like OHCA is a time-sensitive emergency and so timelier detection and improved sensitivity during triage could have a beneficial clinical impact (Green et al., 2019; Muller & Brejnebøl, 2019). Additional studies are recommended in the use of NLP within ambulance control rooms to improve voice recognition. Joseph et al. (2020) undertook research centred on NLP to identify critically ill patients on initial triage in the ED. Critically ill patients deteriorate over a period of time and it is often difficult to identify at the beginning of the presentation of key signs. It is arguable that certain symptoms including functional incapacity, breathlessness and confusion are all subtle signs of many illnesses individually but in aggregate describe the scene of a critically ill patient.

## Discussion

NLP or knowledge detection aims to simulate human reasoning and, like most modern technologies, through its construction it has generated a level of caution and scepticism. Jiang et al. (2017) in a review stated that NLP is of benefit where human actions are questioned around impartiality, objectivity and potential for error. During emergency calls, call handlers are required to use personal judgement which can lead to error. Ivanov et al. (2021), in a US study based in EDs, focused on how machine learning and NLP could improve triage in the ED to manage workflow effectively and safely. They highlighted a lack of impartiality during the nurse ED triage that is related to extrinsic aspects such as time constraints, service demand and emotional distress but also subjective issues such as personal fatigue or simply hunger. Ivanov et al. (2021) found that the use of machine learning removed subjective factors as it acted independently of ED nurses.

Blomberg et al. (2019, 2021) contended that the bias of the human dispatcher was considered when observing non-compliance situations, with some users ignoring the machine-learning alert to support their decision making. This was also identified as a limitation in a study conducted by Berdowski et al. (2009). The authors of this study found that compliance to strict protocols was essential in eliciting the highest sensitivity but it came with the consequence of high false positive rates. It is of interest to note that it may appear that machine learning should reduce elements of human error, but the studies did not address the error potential of the machine-learning algorithm and specifically how issues such as noise and conflicting or unclear information can impact its ability to perform. On balance, it is arguable that all types of error need to be fully considered and reviewed with the introduction of any new technology.

There is a collective view from the literature that machine learning and NLP should be used together to strengthen clinical decision-making but not as a standalone tool. There is further agreement (Muller & Brejnebøl, 2019) in the proposal that machine learning should enhance dispatchers’ clinical decisions rather than be adopted as a singular and isolated tool. It is also proposed (Tootooni et al., 2019) that it would be inappropriate to use machine learning as a replacement for a clinical assessment by doctors and nurses within the ED. This is due to the triage process in any environment having a degree of under- and over-triage, meaning some patients will be considered more urgent than they should and others less urgent, which it could be argued may result in poorer outcomes for patients (Wang et al., 2020). It would seem that it is not realistic to expect one system to provide the perfect triage all of the time; however, combined systems rather than standalone ones may prove more effective and it is prudent to consider how they could be used to identify additional life-threatening situations in the future.

### Limitations

In conducting this review, the authors have tried to remain objective and avoid potential for bias. Given the call handler background of one of the authors, it was essential that this experience was utilised to ensure balance and avoid leading the discussion. The authors strived to ensure that the selection and deselection of articles was based on alignment with the purpose of the literature review and that objectivity was maintained when interpreting findings and identifying and discussing cross-cutting themes.

## Conclusion

From this discussion, it can be argued that NLP and machine learning in the emergency pre-hospital phase of healthcare has the potential to bring enhanced benefits to clinical decision-making. Increased speed in the recognition of cardiac arrest and agonal breathing will remain priorities in the pre-hospital setting, as time saved in commencing CPR is an obviously significant indicator in improving patient recovery. There is a level of enthusiasm to explore the use of NLP and machine learning in acute stroke and critical illness. Critical illness remains an area where early deterioration of signs and symptoms can be subtle and only identified when marked deterioration has occurred. Sepsis is widely reported in the media as a silent killer, so further exploration as to how this treatable life-threatening condition could be identified earlier through technology would be especially welcomed. Although some clinicians remain sceptical that they may be replaced by machines, the evidence suggests that a collaborative approach with a clinician-led machine model would be the optimal balance in safely reducing bias and ensuring greater consistency and accuracy.

## Author contributions

All contributions to this work were conducted by the named authors. KG acts as the guarantor for this article.

## Conflict of interest

None declared.

## Ethics

Not required.

## Funding

None.
